# Angular Displacement and Velocity Sensors Based on Coplanar Waveguides (CPWs) Loaded with S-Shaped Split Ring Resonators (S-SRR)

**DOI:** 10.3390/s150509628

**Published:** 2015-04-23

**Authors:** Jordi Naqui, Jan Coromina, Ali Karami-Horestani, Christophe Fumeaux, Ferran Martín

**Affiliations:** 1 CIMITEC, Departament d’Enginyeria Electrònica, Universitat Autònoma de Barcelona, 08193 Bellaterra, Barcelona, Spain; E-Mails: jan.coromina@e-campus.uab.cat (J.C.); ferran.martin@uab.cat (F.M.); 2 School of Electrical & Electronic Engineering, The University of Adelaide, Adelaide SA 5005, Australia; E-Mails: Ali.K.Horestani@ieee.org (A.K.-H.); christophe.fumeaux@adelaide.edu.au (C.F.)

**Keywords:** split ring resonators, coplanar waveguide, rotation sensors, angular velocity sensors, metamaterial transmission lines

## Abstract

In this paper, angular displacement and angular velocity sensors based on coplanar waveguide (CPW) transmission lines and S-shaped split ring resonators (S-SRRs) are presented. The sensor consists of two parts, namely a CPW and an S-SRR, both lying on parallel planes. By this means, line-to-resonator magnetic coupling arises, the coupling level being dependent on the line-to-resonator relative angular orientation. The line-to-resonator coupling level is the key parameter responsible for modulating the amplitude of the frequency response seen between the CPW ports in the vicinity of the S-SRR fundamental resonance frequency. Specifically, an amplitude notch that can be visualized in the transmission coefficient is changed by the coupling strength, and it is characterized as the sensing variable. Thus, the relative angular orientation between the two parts is measured, when the S-SRR is attached to a rotating object. It follows that the rotation angle and speed can be inferred either by measuring the frequency response of the S-SRR-loaded line, or the response amplitude at a fixed frequency in the vicinity of resonance. It is in addition shown that the angular velocity can be accurately determined from the time-domain response of a carrier time-harmonic signal tuned at the S-SRR resonance frequency. The main advantage of the proposed device is its small size directly related to the small electrical size of the S-SRR, which allows for the design of compact angular displacement and velocity sensors at low frequencies. Despite the small size of the fabricated proof-of-concept prototype (electrically small structures do not usually reject signals efficiently), it exhibits good linearity (on a logarithmic scale), sensitivity and dynamic range.

## 1. Introduction

Transmission lines loaded with electrically small resonators, such as split ring resonators (SRRs) or complementary split ring resonators (CSRRs), have been applied to the implementation of metamaterial-based or metamaterial-inspired circuits where dispersion and impedance engineering play a key role in their designs [1]. Such lines are designated as metamaterial transmission lines. In other applications, the resonance phenomenon is the key aspect. Transmission lines loaded with electrically small resonators formerly used for the implementation of metamaterials, with functionality based on particle resonance (rather than on impedance/dispersion engineering), are usually referred to as transmission lines with metamaterial loading [2]. These structures have been applied to the design of planar bandstop and notch filters [3,4], multiband printed dipole and monopole antennas [5,6,7], common-mode suppressed differential lines [8], radiofrequency barcodes [9], and microwave sensors [10,11,12,13,14,15,16,17,18], among others.

The focus in this work is placed on microwave sensors, where the sensing principle is typically based on the variation of the resonance frequency of the metamaterial resonator loading the line with the variable to be sensed [10,11,12,13,14,15,16,17]. The performance (e.g., sensitivity, linearity and dynamic range) of these sensors is in general good, but it may be degraded by environmental factors (such as temperature and humidity). In other words, these sensors may suffer from non-negligible cross-sensitivities [19], and calibration is likely to become required, unless the measurements are performed on a differential scheme [20]. Recently, a novel approach for the implementation of microwave sensors, based on the disruption of symmetry in transmission lines loaded with electrically small resonators, was proposed [21]. In this strategy, which is considered here and which may be referred to as coupling-modulated resonance, the sensors are designed to be symmetric in the non-actuated (unperturbed) state. When this symmetric configuration is disrupted, such symmetry disruption can be detected from the response of the line to a feeding signal. Commonly, the sensors are designed so that the host transmission line is transparent in the non-actuated (symmetric) state, whereas a notch in the transmission coefficient arises when symmetry is broken [21,22,23,24,25,26,27,28,29]. This functionality is achieved by using the appropriate combination of resonator and line, preventing line-to-resonator coupling if the structure is symmetric. Alternatively, the sensors can be designed with frequency-splitting resonance in order to exhibit a single transmission zero in the unperturbed (symmetric) state, and two transmission zeros when symmetry is perturbed [30,31]. In this later case, the level of asymmetry determines the frequency distance between such transmission zeros. Since symmetry is not affected by environmental factors, sensors based on symmetry properties are more robust against changes in ambient conditions, and may be of special interest in applications where the sensors are subjected to harsh environmental conditions (e.g., space applications).

**Figure 1:**
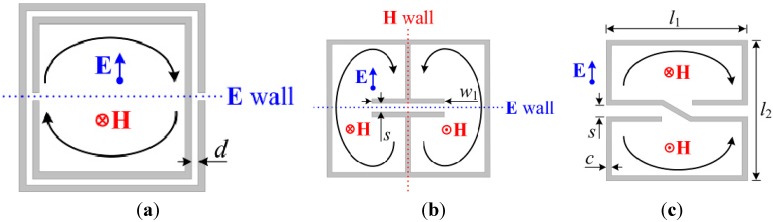
Typical topology of an SRR (**a**), an ELC resonator (**b**), and an S-SRR (**c**). The fundamental resonance is characterized by the orientation of external polarization fields, induced boundary conditions (electric/magnetic walls) at the symmetry planes, and a sketch of the currents.

Another important feature in microwave sensors is their size, as usual in microwave circuits and components. To implement low-cost readout circuits, microwave sensors are sometimes forced to operate at low frequencies. Therefore, transmission line based sensors might not satisfy the size requirements of many applications, unless the sensing elements are electrically very small. In this regard, the S-shaped split ring resonator (S-SRR) [32,33,34], the considered sensing element in this work, is a very convenient resonant element, since it is by far electrically smaller than other resonant elements previously utilized to build up angular displacement and velocity sensors, such as the split ring resonator (SRR) [35] or the electric-LC (ELC) resonator [36]. All of these resonators, whose typical topology is depicted in Figure 1, consist of an arrangement of two loops. Another common characteristic is that they have been proposed in the context of metamaterials, where electrically small elements are mandatory to satisfy homogenization conditions. In contrast, it is important to stress that the resonators considered in Figure 1 differ in regards of their symmetry properties, as the SRR and the ELC are symmetric and bisymmetric, respectively, whereas the S-SRR does not exhibit a symmetry plane.

It will be shown that by using S-SRR-loaded CPW transmission lines, with the S-SRR etched on a movable substrate, it is possible to implement low-frequency angular sensors obtaining simultaneously compact size and competitive performance. This builds upon a preliminary design presented in [37]. Further decrease in the electrical size is possible by means of a broadside-coupled S-SRR (BC-S-SRR), namely a pair of tightly coupled S-SRR arranged face-to-face and rotated 180° to one another [38]. However, this topology is disregarded here because sensor performance is expected to be degraded to a considerable extent (an extremely low attenuation is expected due to the excessive increase in the resonator capacitance produced by the broadside electric coupling).

## 2. The Proposed Topology and Principle of Operation

It is well known that the fundamental resonance of SRRs can be magnetically as well as electrically excited. On the one hand, the magnetic field is needed to be polarized axially to the rings. On the other hand, the electric field requires to be oriented across the symmetry plane of the resonator. Considering the ELC resonator, at the fundamental resonance, the currents in the two loops of the particle flow in opposite directions (one is clockwise while the other counterclockwise). Therefore, the ELC cannot be excited by a uniform time-varying magnetic field orthogonal to the plane of the particle. By contrast, it can be driven by a uniform electric field polarized in the plane of the particle, in the direction orthogonal to the gap (this particle was indeed proposed in [36] for the implementation of resonant-type negative permittivity metamaterials). However, the ELC resonator can also be excited at the fundamental resonance by counter magnetic fields applied to the pair of loops, as Figure 1b illustrates. Similarly, the S-SRR can be excited by counter magnetic fields, since the current flows in opposite directions (clockwise and counterclockwise) at each loop at the fundamental resonance. Since the current flows along the whole resonator length, it follows that the S-SRR is electrically much smaller than the ELC, and even smaller than the SRR, as will be demonstrated shortly.

**Figure 2:**
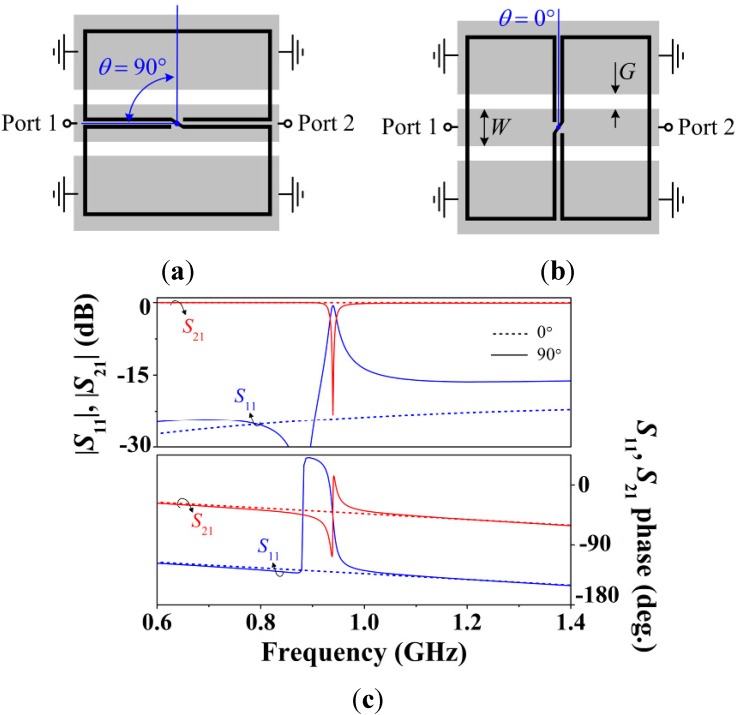
CPW loaded with an S-SRR for (**a**) 90° and (**b**) 0° angular orientations, and (**c**) lossless transmission and reflection coefficients. The angular orientation between the CPW and the S-SRR is determined by the angle 0° ≤ *θ* ≤ 90°, where the S-SRR center is taken as the rotation axis crossing the CPW axis. CPW dimensions are: *W* = 2 mm and *G* = 0.78 mm (50-Ω line). S-SRR dimensions, as denoted in Figure 1, are: *c* = *s* = 0.2 mm and *l*_1_ = *l*_2_ = 10 mm. The substrate is Rogers RO3010 with thickness *h* = 1.27 mm and dielectric constant *ε_r_* = 11.2.

Let us now consider a CPW transmission line loaded with an S-SRR for the two orthogonal and canonical S-SRR orientations, as represented in Figure 2a,b. For the 90° orientation (Figure 2a), the magnetic field lines generated by the line have opposite directions in the individual loops. Accordingly, the particle is excited, perturbing the transmission coefficient magnitude in the form of a transmission notch (or zero), as shown in Figure 2c. Conversely, for the 0° orientation (Figure 2b), even though the cancellation of magnetic field components inside the S-SRR loops is not total, there is in general a negligible net magnetic flux through the loops of the particle, and excitation at the fundamental resonance is prevented provided the particle is small in terms of wavelength. Therefore, the resonator is not coupled to the line for *θ* = 0°, whereas maximum coupling arises when *θ* = 90°. Between these two extreme situations, the frequency response undergoes a perturbation that depends on the coupling level, which in turn is determined by the angular orientation of the resonator. Regardless of the S-SRR orientation, the coupling mechanism between the particle and the CPW is magnetic (obviously with the exception of 0° where there is no effective coupling).

**Figure 3:**
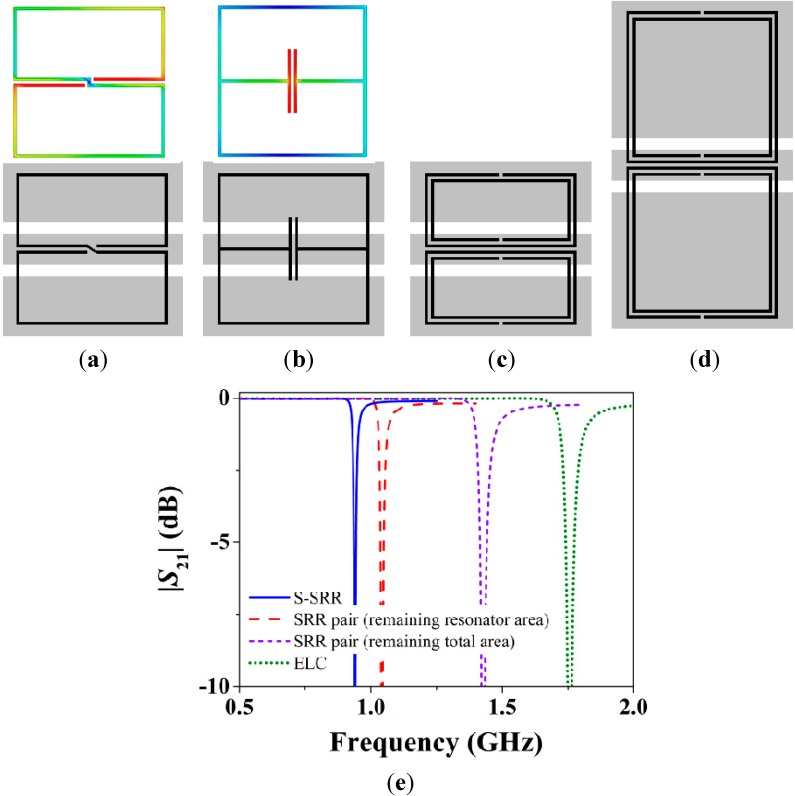
Miniaturization comparison between S-SRRs, ELCs, and SRRs. A CPW is loaded with (**a**) an S-SRR; (**b**) an ELC; (**c**) a pair of SRRs keeping the same total area; and (**d**) a pair of SRRs keeping the same resonator area; (**e**) Magnitude of the lossless transmission coefficient. In (a) and (b) the electric field distributions at the fundamental resonance are plotted.

In order to gain insight into the degree of miniaturization, Figure 3 shows the topology and transmission coefficient of CPWs loaded with the aforementioned resonators. Clearly, for a given resonator area, the lowest resonance frequency is provided by the S-SRR, yet at the expense of the weakest resonance. The benefits of the smaller electrical size are especially noticeable when a pair of SRRs is replaced with a single S-SRR occupying the same total area [39]. It is also remarkable that the resonance frequency of the S-SRR is even smaller than that of a pair of SRRs keeping the same individual resonator area, *i.e.*, with a doubled total size. In the light of these results, the S-SRR is found to be a very attractive resonator in miniaturized CPW-based designs [39].

In the presence of losses (as in all practical situations), the attenuation level in the vicinity of the fundamental S-SRR resonance is determined by the coupling strength, that in turn depends on the relative orientation between the line and the S-SRR. Therefore, the proposed structure can be used for sensing the rotation angle between the CPW transmission line and the S-SRR. Furthermore, by using time response from the angular displacement sensor, the angular velocity can be readily inferred, as will be shown later.

As reported in [26,27], the principle for rotation sensing in ELC-loaded CPWs is based on symmetry properties. By aligning the symmetry plane of the line (a magnetic wall) with the electric wall of the bisymmetric resonator, line-to-resonator coupling is prevented. By contrast, when the magnetic wall of the resonator is aligned with the CPW symmetry plane, the two elements are tightly coupled. Hence, whereas the ELC-loaded line is transparent for the 0° orientation, it exhibits significant attenuation for the 90° orientation. For intermediate orientations, the coupling is angle-dependent. The essential benefit of the ELC as a sensing element is the fact that it exhibits two symmetry planes of distinct nature, an electric wall and a magnetic wall. On the other hand, high miniaturization levels cannot be achieved by the ELC resonator. In this paper, it is demonstrated that, by sacrificing symmetry, the S-SRR is a suitable choice towards miniaturization-oriented sensors with competitive performance. Thus, even though the S-SRR does not exhibit any symmetry plane, S-SRR-and ELC-loaded CPWs behave similarly, and the sensing principle of both structures is identical. Nevertheless, the penalty of using a non-symmetric resonator is that a notch for 0° might appear if a sufficient small amount of magnetic field flux illuminates the resonator as a result of a non-absolute field cancellation. Accordingly, the designer must be cautiously aware of this issue.

## 3. Equivalent Circuit Model and Parameter Extraction

An equivalent circuit model of CPWs loaded with arbitrarily angular-oriented ELC resonators was proposed in [27]. It was shown that, as a first-order tendency, the net mutual inductance between the line and resonator was the only angle-dependent circuit element. In short, for *θ* = 90° the mutual magnetic coupling is maximum, whereas for *θ* = 0° there is no net mutual coupling.

**Figure 4:**
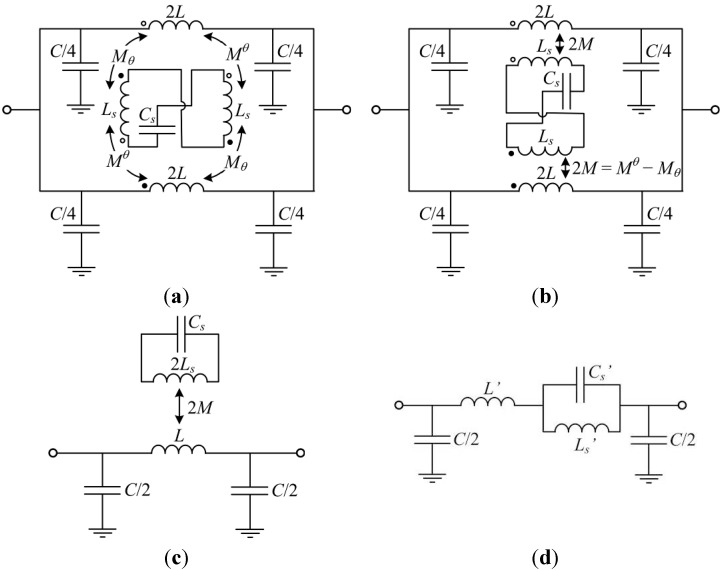
Proposed lossless angle-dependent equivalent circuit model of an S-SRR-loaded CPW. Model with the CPW symmetrically split (**a**) where different magnetic coupling mechanisms are identified and (**b**) where an effective magnetic coupling is considered; (**c**) Simplified model and (**d**) equivalent transformed model. Further details on the derivation of the original circuit models corresponding to an ELC-loaded CPW can be found in [27].

A similar circuit applies to S-SRR-loaded CPWs, as depicted in Figure 4a. For a fixed 90° orientation, this circuit is equivalent to that reported in [39]. The inductance of each loop is represented by *L_s_*, while the resonator capacitance is modeled by *C_s_*. The CPW is divided into two identical halves, with *L* and *C* being the CPW inductance and capacitance, respectively. Finally, each CPW half is magnetically coupled to each loop through the complementary angle-dependent mutual inductances *M_θ_* and *M^θ^* (see Figure 6b). By defining a net mutual inductance, *M*, the circuit in Figure 4b is obtained. This later circuit can be simplified to an equivalent one represented in Figure 4c, which in turn can be transformed to that depicted in Figure 4d using the following equivalences [40]:

(1) $$C_{s}´=\frac{L_{s}}{2{\left(\omega_{0}M\right)}^{2}}=C_{s}{\left(\frac{L_{s}}{M}\right)}^{2}$$

(2) $$L_{s}´=4C_{s}{\left(\omega_{0}M\right)}^{2}=2\frac{M^{2}}{L_{s}}$$

(3) $$L´=L-L_{s}´$$

where the angular resonance frequency is:

(4) $$\omega_{0}=\frac{1}{\sqrt{2C_{s}L_{s}}}=\frac{1}{\sqrt{C_{s}´L_{s}´}}$$

It is essential to point out that, when *θ* = 0°, the proposed circuit aimed at sensing purposes should ideally provide *M* = 0. Since this cannot be absolutely satisfied using non-symmetric structures (e.g., S-SRRs as loading elements), in practice the resulting *M* should be close to zero as much as possible. Otherwise a notch, although shallow, is likely to appear.

**Figure 5:**
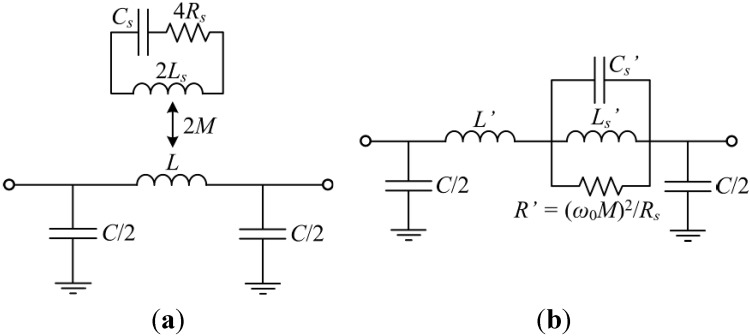
Lossy angular-dependent equivalent circuit model of an S-SRR-loaded CPW. (**a**) Simplified model and (**b**) equivalent transformed model.

In real bandstop structures losses always prevent the attenuation peak from going to infinity. Since the electrical variable to be sensed in the proposed approach is the amplitude of the transmission notch, losses are a factor determining the sensor performance. To analyze losses, let us assume that resonator-related losses are the main source of power loss. Following the terminology in [27] as earlier, losses are accounted for by a resistor of 2*R_s_* corresponding to each half of the resonator. Hence, the circuits in Figure 5 are obtained, which allow for the modeling including losses. It is noted that under lossless conditions, *R_s_* is null while *R'* is infinite, leading to the representation of Figures 4c,d.

**Figure 6:**
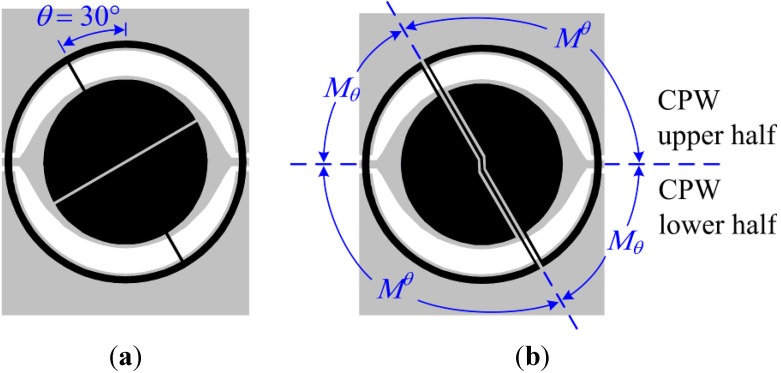
Illustration of resonator topology transformation from (**a**) an ELC resonator [27] to (**b**) an S-SRR. The geometrical division associated to the mutual inductances corresponding to the two CPW halves and the two S-SRR loops is sketched in (b). The substrate is Rogers RO3010 with thickness *h* = 1.27 mm, relative permittivity *ε_r_* = 11.2, and loss tangent tan*δ* = 0.0023. Dimensions are: for the line, *W* and *G* are tapered so that the characteristic impedance is 50 Ω (the radial distance from the center of the CPW to the lateral ground planes is fixed to 7.6 mm); for the resonators: external loop mean radius *r*_0_ = 8.05 mm, circular patch outer radius *r*_1_ = 5.6 mm, *c*_1_ = *s* = 0.2 mm, and *c*_2_ = 0.5 mm.

With the purpose of validating the circuit models, the ELC-loaded CPW shown in Figure 6a is considered as a baseline [27]; this structure was seen to exhibit good linearity of the notch magnitude with the rotation at a roughly constant resonance frequency (attenuation is characterized in dB as usual). Good linearity was explained to be due to the circularly-tapered CPW in combination with the circularly-shaped ELC. A constant resonance frequency, on the other hand, was attributed to invariant resonator inductance and capacitance with rotation. As a next step, the ELC topology is modified so that an S-SRR is obtained, as depicted in Figure 6b. Thereby, similar capacitance and loop inductances for both resonators are expected (*C_s_* ≈ *C_e_* and *L_s_* ≈ *L_e_*, where the subscripts *s* and *e* stand for the S-SRR and ELC resonator, respectively), allowing for an approximate quantitative comparison.

Prior to verifying the circuit model, let us first derive approximate analytical relationships between ELC- and S-SRR-loaded CPWs by assuming that *C_s_* ≈ *C_e_* and *L_s_* ≈ *L_e_*. Despite the fact that the loop inductances are assumed to be similar, while the two ELC loops are connected in parallel [27], those of the S-SRR are connected in series (Figure 4). Hence, the corresponding total inductances are *L_e_*/2 and 2*L_s_*, and thus, by converting the ELC into an S-SRR the total inductance is expected to increase by roughly four times. In consequence, S-SRR-loaded lines are expected to be electrically smaller by a factor of two approximately. Indeed, according to the circuit model reported in [27] the resonance frequency of an ELC-loaded line is *ω*_0*e*_ = (*C_e_L_e_*/2)^−1/2^. On the other hand, with reference to the circuit model of an S-SRR-loaded line, the resonance frequency is *ω*_0*s*_ = (2*C_s_L_s_*)^−1/2^, satisfying *ω*_0*s*_ ≈ *ω*_0*e*_/2. However, the frequency downshift provided by the S-SRR is achieved at the expense of suffering from a lower ratio *L_s_*'/*C_s_*' of the equivalent parallel resonator (4*L_s_*'/*C_s_*' ≈ *L_e_*'/*C_e_*' since *L_s_*' ≈ *L_e_*' and *C_s_*' ≈ 4*C_e_*'), which is intimately related to the resonance bandwidth (bandwidth broadens with an increase in *L_s_*'/*C_s_*'). Specifically, the unloaded quality factor at resonance [41] is:

(5) $$Q_{u}=R´\sqrt{\frac{C_{s}´}{L_{s}´}}=\frac{1}{2\sqrt{2}R_{s}}\sqrt{\frac{L_{s}}{C_{s}}}$$

the fractional bandwidth at +3 dB being approximately *FBW_u_* = 1/*Q_u_* (the higher the *Q_u_*, the more accurate the approximation) [41]. Regarding the corresponding absolute bandwidth, *BW_u_* = *f*_0_/*Q_u_*, this gives:

(6) $$BW_{u}=\frac{1}{2\pi R´C_{s}´}=\frac{R_{s}}{\pi L_{s}}$$

On the other hand, the frequency dependence of the resistance [41] is taken into account by expressing the resistances in terms of the resonance frequency as *R_s_* = *k_s_ω*_0*s*_^1/2^ and *R_e_* = *k_e_ω*_0*e*_^1/2^, where *k* is a constant dependent on geometrical and material parameters. Since *k_s_* ≈ *k_e_*, one finds that *R_s_* ≈ *R_e_*/2^1/2^ and *R_s_'* ≈ *R_e_'*/2^3/2^, meaning that the notch depth in S-SRR-based CPWs cannot be superior to that in ELC-loaded lines, as will be shown throughout this work. It is also found that *BW_us_* ≈ *BW_ue_*/2^1/2^, *FBW_us_* ≈ 2^1/2^ *FBW_ue_* and *Q_us_* ≈ *Q_ue_*/2^1/2^.

To perform the circuit modeling validation, the focus is only on the 90° orientation, owing to the fact that this is the most representative case to gain insight into the level of line-to-resonator coupling. For such an orientation, the extracted circuit parameters of the circuit in Figure 4d for the S-SRR-loaded line of Figure 6b are listed in Table 1 (the parameters for the ELC-loaded line are reproduced from [27]). The extraction has been carried out by means of the methodology reported in [40] based on the *S* parameters obtained by lossless electromagnetic simulation. Afterwards, the parameters of the model in Figure 4c are obtained by the transformation Equations (1)–(3) using an estimated value of the loop inductance of the resonators (see Table 1). To perform such an estimation, the procedure reported in [27] is used; the inductance seen looking into the loop terminals is inferred by electromagnetic simulation in the absence of the capacitive patches and the CPW. The estimated inductances in terms of the individual loops are *L_e_* = 25.6 nH and *L_s_* = 22.2 nH. As expected, although these inductances are relatively similar, the resonance frequency is reduced by roughly half, namely from *f*_0*e*_ = 803 MHz to *f*_0*s*_ = 388 MHz. As mentioned before, in order to characterize the notch magnitude, losses need to be taken into account. To this end, the resistances in Figure 5 are extracted by curve fitting the circuit simulation to the lossy electromagnetic simulation (Table 1). Table 1 also indicates the unloaded *Q*, namely *Q_u_*, and the resulting *Q*-factor of the transmission notch, *i.e.*, *Q* = 1/*FBW* = *f*_0_/*BW* (where *FBW* and *BW* are the fractional and absolute bandwidths at +3 dB with respect to the maximum attenuation). The resulting relationships between the considered S-SRR- and ELC-based structures can be seen in Table 2, which are in reasonable accordance with the analytical derivation.

**Table 1:** Extracted circuit elements of the models in Figure 5 for the ELC- and S-SRR-loaded CPWs of Figure 6 with 90° orientation.

	*C* (pF)	*L* (nH)	*C_s_* (pF)	*L_s_* (nH)	*M* (nH)	*R_s_* (Ω)	*k* Ω·(rad/s)^−1/2^	*L'* (nH)	*C_s_'* (pF)	*L_s_'* (nH)	*R'* (Ω)	*f*_0_ (MHz)	*Q_u_*	*Q*
S-SRR	5.01	7.11	3.79	22.20	2.84	0.37	7.5 × 10^−6^	6.38	231.33	0.73	129.8	388	73	59
ELC	5.59	6.40	3.07	25.60	2.72	0.49	6.9 × 10^−6^	5.82	67.67	0.58	385.1	803	132	129

**Table 2:** Most relevant relationships between S-SRR- and ELC-loaded CPWs.

	*L_e_/L_s_*	*M_s_*/*M_e_*	*R_e_*/*R_s_*	*k_s_*/*k_e_*	*R_e_'*/*R_s_'*	*f*_0*e*_/*f*_0*s*_
Analytically assuming *C_s_* = *C_e_* and *L_s_* = *L_e_*	1	1	1.41	1	2.83	2
Extracted circuit parameters in Table 1	1.15	1.04	1.32	1.09	2.97	2.07

**Figure 7:**
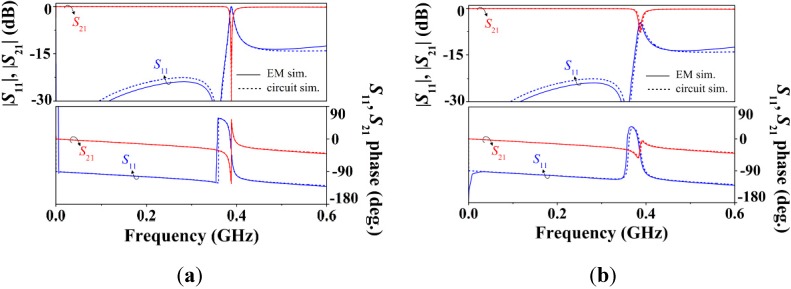
Frequency response of the S-SRR-loaded line of Figure 6b for 90° orientation obtained by (**a**) lossless and (**b**) lossy electromagnetic and circuit simulations. The circuit elements are indicated in Table 1. Despite its frequency dependence, a constant value resistance computed at the resonance frequency is assumed (*R_s_* = *k*_*s*_ω_0*s*_^1/2^).

The comparison of the electromagnetic and circuit simulations corresponding to the S-SRR-loaded line is plotted in Figure 7, where good agreement is apparent (for this angular orientation, lossless conditions, and a uniform CPW, it has been previously shown in [39] that the circuit model is able to fit accurately the structure response). As compared to ELC-loaded CPWs [27], since the electrical size at resonance is decreased by the S-SRR, the bandwidth of validity of the circuit model in S-SRR-loaded lines is broadened. This enhancement is a further indicator on the achievable small electrical size and an additional advantage of the S-SRR.

In summary, when transforming an ELC to an S-SRR, despite the fact that the individual circuit parameters in Figure 5a are similar for both cases, the equivalent parameters in Figure 5b are moderately different as a result of the dual connection (series or parallel) of the two loops forming the resonators. Therefore, apart from symmetry-related differences between the ELC resonator and the S-SRR, another fundamental distinct feature derived from the analysis of their equivalent circuit models is the total inductance and associated resistance provided by them, which have a direct impact on the attenuation characteristics in the vicinity of resonance. It is worth mentioning that this strategy based on changing the resonator topology is inversely analogous to that carried out in [8] where a CSRR is converted into a double slit CSRR (DS-CSRR). These resonators are symmetric and bisymmetric, respectively, the latter providing essentially four times smaller inductance. Thereby, the rejection bandwidth widens as desired, although the resonance frequency obviously increases.

## 4. Performance Tradeoffs

The circuit models in Figure 5 can be viewed as the general model of a transmission line coupled magnetically to an electromagnetic resonator. The present section is intended to demonstrate that the frequency and magnitude of the transmission notch (two fundamental sensor parameters) for fixed physical dimensions, are closely related. The analysis that is presented complements other studies related to the optimization and limitations of the bandstop functionality exhibited by the considered structures [42,43].

**Figure 8:**
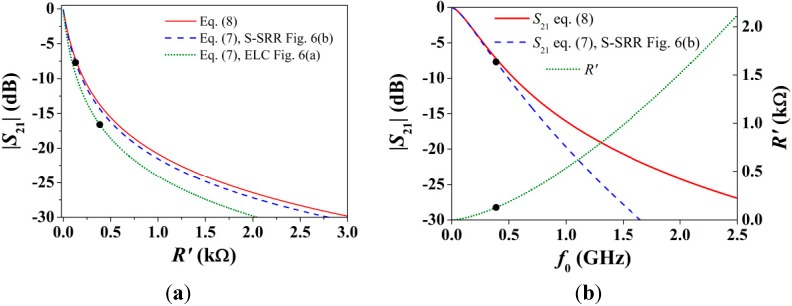
(**a**) Notch magnitude when sweeping *R'*; (**b**) Notch magnitude and *R'* when sweeping *f*_0_. The circuit parameters are those indicated in Table 1, and *Z*_0_ = 50 Ω. The specific operating points in Table 1 are also mapped.

As drawn earlier, since losses increase with frequency, the S-SRR benefits from a smaller series resistance (*R_s_* ≈ *R_e_*/2^1/2^) which directly influence the equivalent parallel resistance *R'*. However, as a result of the frequency downshift, *R'* is reduced rather than increased (*R_s_'* ≈ *R_e_'*/2^3/2^), and the notch depth in the topologies of Figure 6 drops from −16.6 dB [27] to −7.7 dB. Analytically, the notch magnitude in the circuit of Figure 5b can be expressed as:

(7) $$\left|S_{21}\right|(\text{dB})=20{\text{log}}_{10}\left|\frac{1}{Z_{s}\left(\frac{Y_{p}^{2}Z_{0}}{2}+\frac{1}{2Z_{0}}\right)+Y_{p}\left(Z_{s}+{Ζ}_{0}\right)+1}\right|$$

where *Z*_s_ is the impedance of the series branch, *Y_p_* is the admittance of the shunt branch, and *Z*_0_ is the port impedance. According to Equation (7), the equivalent line elements in the presence of the S‑SRR (*L'* and *C*) influence the notch depth. Nevertheless, for electrically small structures, the main signal rejection mechanism is due to *R'* rather than to the line elements. In the absence of these line elements (*Z*_s_ = *R'* and *Y_p_* = 0), the notch magnitude reduces to:

(8) $$\left|S_{21}\right|(\text{dB})=20{\text{log}}_{10}\left|\frac{1}{1+R´/2{Ζ}_{0}}\right|$$

imposing a boundary dictating the minimum attenuation. Specifically, at very low frequencies, where the line elements do not play a significant role, Equation (7) converges to Equation (8). Numerical solutions of the nonlinear Functions (7) and (8) where *R'* is swept are plotted in Figure 8a. For the S-SRR-loaded line, due to the smaller electrical size, Equation (7) is closer to Equation (8) than for the ELC-loaded line. As can also be noticed, in the lower region of *R'*, the notch magnitude is very sensitive to *R'*, suggesting that a relatively small minimum *R'* must be fulfilled to obtain a reasonable attenuation (which numerically could be fixed by specifications). However, Figure 8a provides no insight into the dependence of |*S*_21_| with *f*_0_ through *R'*, *i.e.*,

(9) $$R´=\frac{{\left(\omega_{0}M\right)}^{2}}{R_{s}}=\frac{M^{2}}{k}\omega_{0}^{3/2}$$

To this end, Figure 8b shows |*S*_21_| and *R' versus* *f*_0_, assuming that *M* and *k* are constant (this condition makes sense, for instance, when comparing S-SRR- and ELC-loaded CPWs with the same dimensions). The key aspect is that, the lower the resonance frequency, the weaker the attenuation, so that decreasing *f*_0_ (or equivalently the electrical size) invariably degrades |*S*_21_|. In other words, there is a tradeoff between lowering the resonance frequency and enhancing the attenuation, and it is explained through *R'*.

In addition, let us now analyze the bandwidth at +3 dB of the unloaded resonator given by Equation (6), which in terms of frequency can be rewritten as:

(10) $$BW_{u}=\frac{2R_{s}C_{s}\omega_{0}^{2}}{\pi }=\frac{2kC_{s}}{\pi }\omega_{0}^{5/2}$$

**Figure 9:**
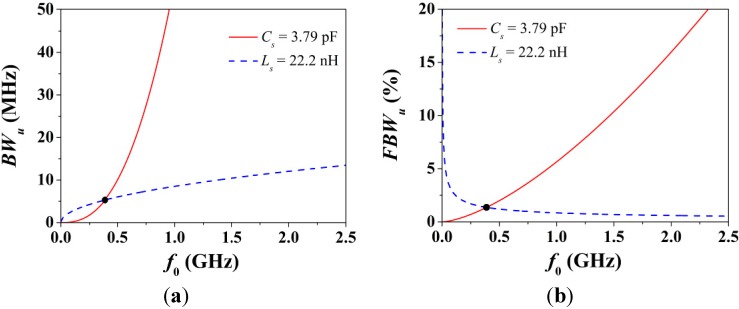
(**a**) Absolute fractional bandwidth at +3 dB of an unloaded resonator given by Equation (10) *versus* the resonance frequency; and (**b**) its fractional bandwidth. The circuit parameters are those of the S-SRR-based structure in Table 1. In order to sweep the resonance frequency, either the capacitance or the inductance is considered to be constant. The specific operating point in Table 1 is also mapped.

Expressions (7)–(10) clearly explain that losses round the attenuation peak off, a well-known consequence of losses. More importantly, if the frequency dependence in *R_s_* is ignored in Equations (9) and (10), although *C_s_* is not decoupled to *f*_0_ in Equation (10), both *R'* and *BW_u_* are quadratically proportional to *f*_0_. Therefore, *BW_u_* and |*S*_21_| are intimately related to one another with respect to *f*_0_, and in the limit where *f*_0_ → 0, both *BW_u_* and |*S*_21_| → 0. To clarify this, Figure 9 shows the dependence of *BW_u_* and *FBW_u_* with *f*_0_. Note from Equation (6) that when *L_s_* is constant, the dependence of *BW_u_* with frequency is only through *R_s_*. Notice also that *BW_u_* narrows regardless of whether *f*_0_ is decreased by increasing the inductance or capacitance. The reason is that the slope of *BW_u_* with frequency is always positive. By contrast, *FBW_u_* depends on the ratio *C_s_*/*L_s_* (property that can be deduced from *Q_u_*), and its slope with frequency is not univocally determined by whether *f*_0_ increases or decreases.

**Figure 10:**
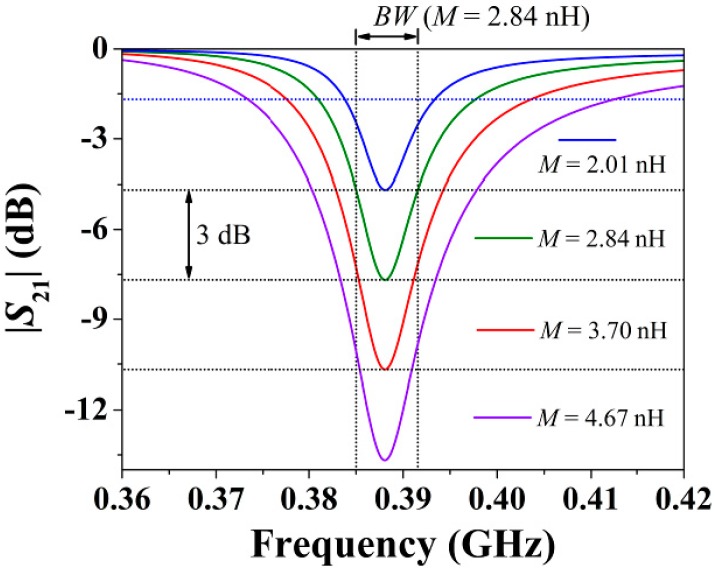
Transmission coefficient (magnitude) of the circuit model in Figure 5a for the circuit parameters of the S-SRR-loaded CPW in Table 1, where *M* is varied such that the notch depth is changed by steps of ±3 dB.

Lastly, it should be highlighted that neither *Q_u_* nor *BW_u_* depends on *M*, whereas |*S*_21_| is strongly influenced by *M*. To illustrate this, Figure 10 plots the transmission coefficient inferred from the circuit model for different values of the mutual inductance. As can be observed, regardless of the value of *M*, the bandwidth of the resonance, *BW* (different from but related to *BW_u_*), is nearly constant when changing *M*. On the contrary, |*S*_21_| changes drastically.

## 5. Sensor Design

This section is devoted to the optimization of S-SRR-loaded CPWs aimed for operation as rotation sensors based on measuring the notch depth and with an angular dynamic range 0° ≤ *θ* ≤ 90°. Sensor performance is determined by the operating frequency *f*_0_, frequency stability *df*_0_/*dθ*, dynamic range (|*S*_21_|*_θ_*_=90°_ − |*S*_21_|*_θ_*_=0°_), sensitivity *d*|*S*_21_|/*dθ*, and linearity (deviation from a constant sensitivity). Since replacing an ELC with an S-SRR results in a lower resonance frequency obtained at the expense of a weaker attenuation notch, the particular objective is to obtain a good balance between *f*_0_ and |*S*_21_|. This means to reduce *f*_0_ as much as possible whilst |*S*_21_| being degraded as less as possible (although *f*_0_ and |*S*_21_| could be dictated by practically determined specifications, instead of imposing fictitious ones, the purpose is here to prove the concept).

**Figure 11:**
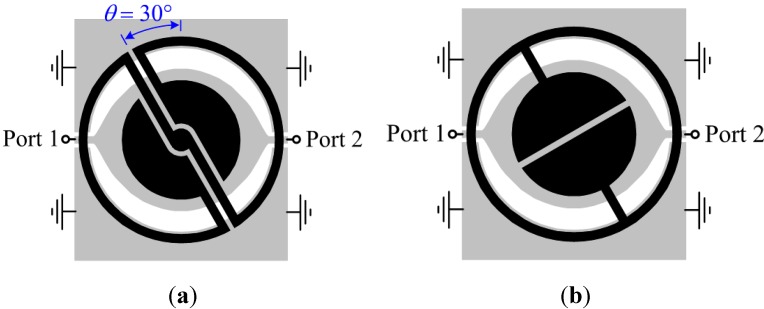
(**a**) Topology of the proposed rotation sensor based on an S-SRR; (**b**) Equivalent macroscopic topology for a sensor based on an ELC for comparison purposes. The CPW dimensions and substrate are the same as those in Figure 6. Resonator dimensions are: external loop mean radius *r*_0_ = 8.175 mm, circular patch outer radius *r*_1_ = 5 mm, *s* = 0.5 mm, and *c* = 0.75 mm.

Though the topology in Figure 6b resonates at a very low frequency, it suffers from a poor attenuation peak, making this topology marginally useful for operation as a sensor. To improve the dynamic range of attenuation, the equivalent resistance *R'* needs to be increased (Figure 8). With a view to achieving this at low frequencies (achieving a good balance between *f*_0_ and |*S*_21_|), the approach chosen is to decrease the term *k* in Equation (9). For this purpose, the topology depicted in Figure 11 is proposed, where the fundamental strategy is to reduce the resonator resistance, *R_s_*, by widening the strips of the loops (losses are primarily due to the inductive narrow strips of the loops). Since this simultaneously reduces the inductance, the resonance frequency shifts upwards, and this could be compensated by increasing the capacitance. However, the patches are kept almost unaltered in order not to degrade the mutual magnetic coupling *M*, another key parameter in *R'* (it is evident that a trivial solution to modify *M* is to change the spacing between the line and the resonator). The extracted circuit elements for the proposed design, listed in Table 3, corroborate the tailoring of these parameters. It should be noted that although *f*_0_ is slightly increased, the enhancement in *R'* is actually mainly due to the drastic decrease in *k*. In short, the extremely small *R'* due to a decrease in *ω*_0_ (produced by an increase of the total inductance) is compensated by decreasing *R_s_* (which in fact decreases the total inductance at the same time), boosting *R'*.

**Table 3:** Extracted circuit elements of the models in Figure 5 for the S-SRR-loaded CPWs of Figure 6 and Figure 11 with 90° orientation.

	*C* (pF)	*L* (nH)	*C_s_* (pF)	*L_s_* (nH)	*M* (nH)	*R_s_* (Ω)	*k* Ω·(rad/s)^−1/2^	*L'* (nH)	*C_s_'* (pF)	*L_s_'* (nH)	*R'* (Ω)	*f*_0_ (MHz)	*Q_u_*	*Q*
Figure 6	5.01	7.11	3.79	22.20	2.84	0.37	7.5 × 10^−6^	6.38	231.33	0.73	129.8	388	73	59
Figure 11	6.03	7.33	3.43	17.48	2.77	0.19	3.5 × 10^−6^	6.45	136.85	0.88	340.4	460	135	129

**Figure 12:**
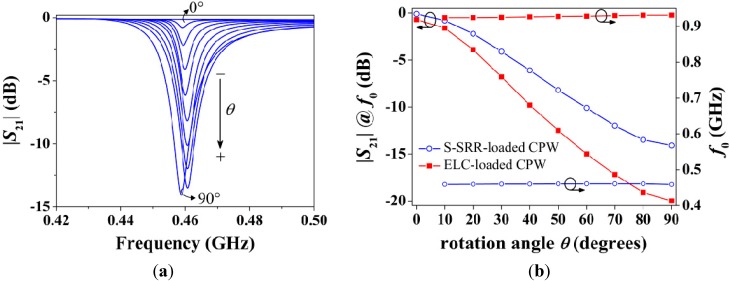
Simulated characteristics of the structures in Figure 11. (**a**) Transmission coefficient magnitude for the S-SRR-loaded CPW; and (**b**) transfer functions as sensors for different discrete angles.

The characterization of the designed structure as a rotation sensor inferred from electromagnetic simulations is given in Figure 12. The transmission coefficients are shown in Figure 12a and the corresponding notch magnitude and frequency *versus* the rotation angle are plotted in Figure 12b. In contrast to the topology in Figure 6, it can be drawn that the proposed geometry exhibits a reasonably good balance between the resonance frequency and the notch depth, since by sacrificing a relatively small notch magnitude (−20 to −14.1 dB for 90°, *i.e.*, 29.5%) the operating frequency is significantly decreased (927 to 460 MHz in average, *i.e.*, 50.4%). Indeed, the notch magnitude between the S-SRR- and the ELC-based structures is not as different as predicted by the circuit model. The reason is attributed to the fact that the accuracy of the circuit model is degraded when the electrical size increases, and the ELC-loaded CPW may be not sufficiently electrically small to allow for an accurate comparison (the relationship *R_s_'* ≈ *R_e_'*/2^3/2^ is no longer accurate, *R_s_'* being closer to *R_e_'*, thus benefiting the S-SRR). Furthermore, a better stability in the resonance frequency with rotation is achieved when the S-SRR is employed. By defining the frequency-detuning bandwidth as ∆*f*_0_ = *f*_02_ − *f*_01_ (where *f*_02_ and *f*_01_ are the maximum and minimum resonance frequencies, respectively), *f*_0_ ranges within a band of ∆*f*_0*e*_ = 7.3 MHz for the ELC-loaded CPW (0.8% with respect to the average resonance frequency), while it varies only by ∆*f*_0*s*_ = 1.9 MHz (0.4%) for the S-SRR-loaded counterpart. These results validate the approach and the usefulness of S-SRR for the implementation of rotation sensors.

## 6. Prototype Characterization

**Figure 13:**
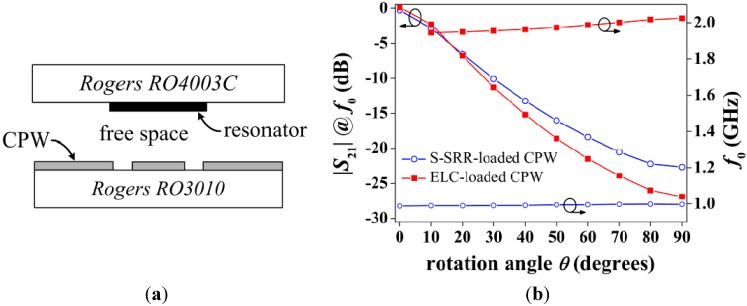
(**a**) Sensor layer cross-section; and (**b**) simulated transfer function for the topologies in Figure 11. The parameters of Rogers RO4003C are: thickness *h* = 0.8128 mm, relative permittivity *ε_r_* = 3.55, and loss tangent tan*δ* = 0.0021.

Thus far, the S-SRR has been etched on the back side of the CPW substrate with the purpose of proving the sensing principle. However, in a real application, the S-SRR must be etched on a substrate different from that of the CPW, allowing for relative motion between both layers. Hence, to complete the performance characterization of S-SRR-loaded lines as rotation sensors, an experimental set-up including an in-between air gap region is considered, which is specifically illustrated in Figure 13a [27]. As can be seen, the S-SRR is etched on a low permittivity substrate to enhance the notch amplitude. It should be highlighted that commercial Rogers substrates are considered with no loss of generality, since the key aspect is merely to employ low-loss substrates to minimize dielectric losses.

The corresponding sensor transfer functions in this stacked substrate-air-substrate using the designs in Figure 11 are plotted in Figure 13b. The combination of the air gap and the low permittivity substrate results in a drastic decrease in the resonator capacitance. As a consequence the resonance frequency suffers from an upwards shift and the notch amplitude strengthens. It is therefore obvious that the S-SRR must be designed in accordance with the substrates considered in order to tune it at the desired frequency. Regarding the notch depth enhancement, a relevant consequence is that a resonance is observed even for 0° orientation. Nevertheless, since the notch depth is extremely weak (very close to 0 dB), the overall dynamic range and sensitivity in amplitude are effectively improved. Furthermore, this undesired resonance points out that the S‑SRR is indeed tightly coupled to the CPW, as required to enhance the attenuation. It is worth mentioning that in this prototype the balance under optimization is improved; |*S*_21_| decreases from −26.9 to −22.7 dB (15.6%) and *f*_0_ decreases from 1.98 to 0.993 GHz (49.9%). With respect to the frequency deviation, the frequency shift for the S-SRR-based structure amounts to only ∆*f*_0*s*_ = 10.4 MHz (1%), which is remarkably better than for the ELC-loaded CPW with ∆*f*_0*e*_ = 79 MHz (4%). Despite the fact that ∆*f*_0_ is degraded in this prototype as compared to the single substrate structure, it is still within acceptable limits.

**Figure 14:**
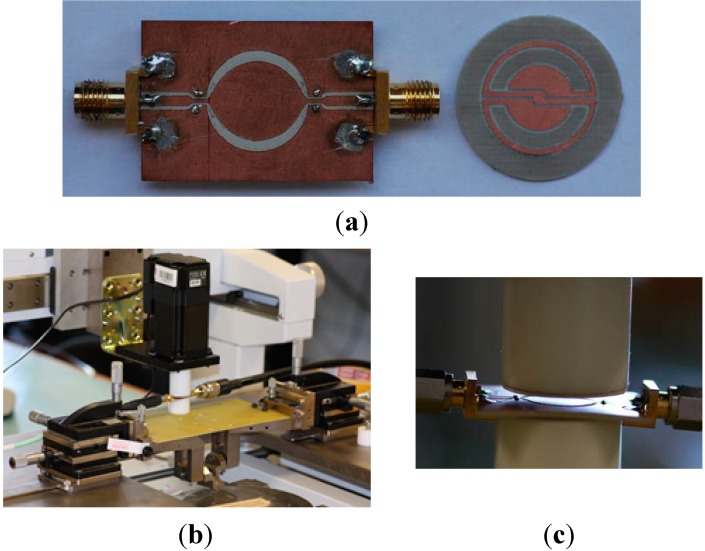
The fabricated prototype (**a**) and experimental set-up (**b**), including details (**c**), for the characterization of the rotation sensor with an STM 23Q-3AN step motor. The parameters of the Teflon cylinder adhered to the step motor are: *h* = 3.5 cm, *ε_r_* = 2.08 and tan*δ* = 0.0004.

**Figure 15:**
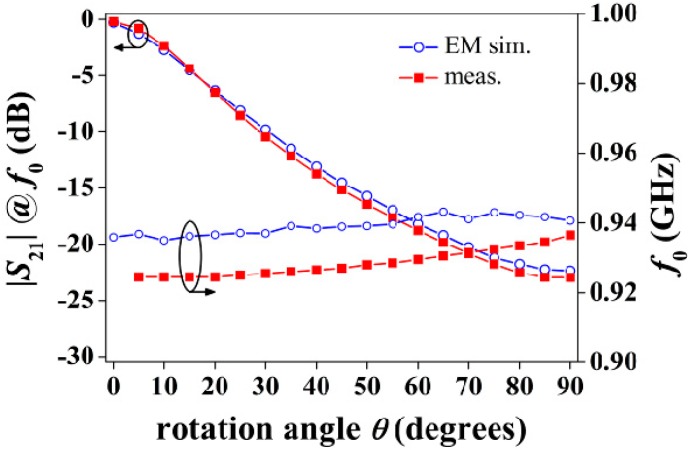
Simulated and measured transfer function of the sensor prototype.

In order to measure the S-SRR-based sensor, input/output transmission line sections are inserted at the designed distance (see Figure 14). Since the CPW is no longer electrically small, and considering that regardless of the S-SRR angular orientation the structure is asymmetric, vias and backside strips are utilized to connect the two lateral CPW ground planes and thus suppress the slot mode,. In the experimental set‑up, the S-SRR substrate is fastened to a Teflon cylinder, which is rotated by the action of a step motor (Figure 14). The simulated (including the Teflon cylinder) and measured transfer functions are shown in Figure 15. It is noted that the notch for 0°, masked by the CPW line insertion loss, was actually not detected in measurements. The measured sensor parameters are: the frequency deviation is ∆*f*_0_ = 12 MHz (1.3%), the output dynamic range is 22.8 dB, the average sensitivity is 0.25 dB/°, and the average linearity is 2.1 dB (9.3%). Therefore, the performance is comparable to those reported in [26,27,28] with the advantage of operating at a considerably lower resonance frequency.

Alternative symmetry-inspired rotation sensors based on notch depth modulation using other resonant particles have been proposed in the last few years [21,23,25], however with considerably limited angular dynamic ranges. This means that rotation speed measurements might be not straightforward. On the other hand, microwave rotation sensors based on frequency [17] and phase shift [18], with higher dynamic range (180° and 360°, respectively) and good linearity have also been recently reported. However, these sensors cannot be extended to the implementation of angular velocity sensors with a readout circuit as simple as with amplitude levels (see next section).

## 7. Angular Velocity Sensor

**Figure 16:**
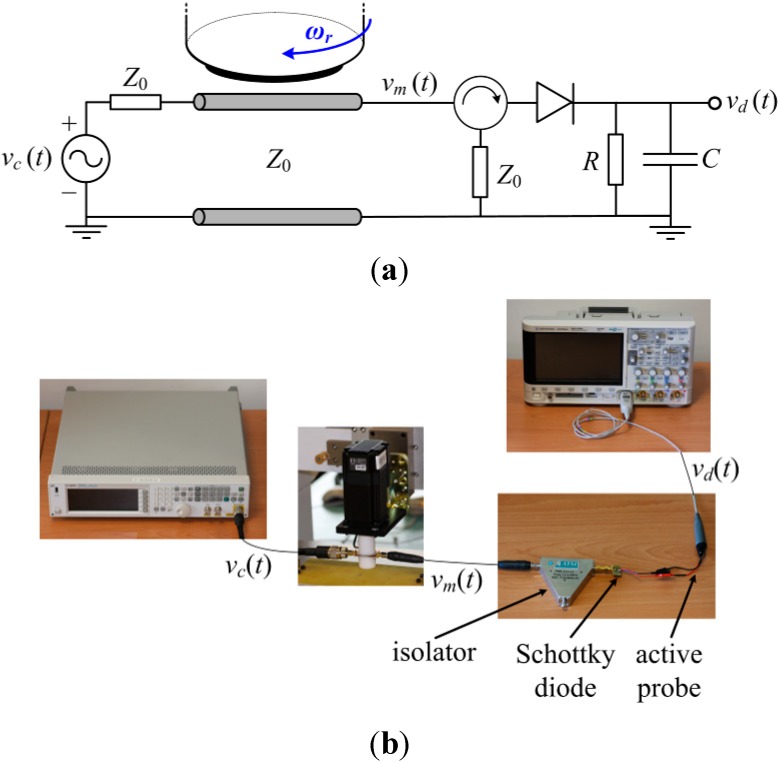
Scheme (**a**) and experimental set-up (**b**) for the measurement of the angular velocity. The carrier signal has been generated by means of the Agilent N5182A signal generator, whereas the output waveform has been recorded by the Agilent 3054A oscilloscope. The isolator has been implemented with the ATM ATc1-2 circulator, with one of the ports terminated with a matched 50-Ω load. The envelope detector is synthesized by the Avago Technologies HSMS-2860 Schottky diode and the single-ended active probe Agilent N2795A (with an input impedance of 1 MΩ in parallel with 1 pF).

**Figure 17:**
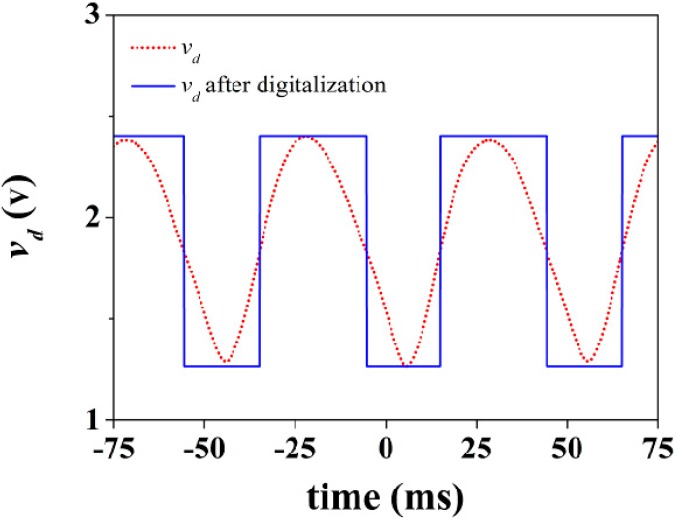
Output waveform for an angular velocity of 600 rpm.

The rotation sensor can be used as an angular velocity sensor using an appropriate readout circuit. One possible means to measure the angular velocity is by cascading a circulator (configured as an isolator to avoid unwanted reflections) and an envelope detector to the rotation sensor. By injecting a carrier signal tuned at the fundamental resonance frequency of the S-SRR, the amplitude of the signal is modulated by the rotating S-SRR. The envelope of the output signal can be then obtained by means of the envelope detector, and hence the angular velocity can be inferred from the time distance between adjacent peaks. Note that the period of the envelope signal corresponds to half a rotation period. Thus, from this simple measurement in time domain, using an oscilloscope connected to the output port of the envelope detector to monitor the output voltage waveform, it is possible to accurately determine the rotation speed. The scheme and photograph of the experimental set-up are depicted in Figure 16 [27]. We have considered an angular velocity of 600 rpm (*f_r_* = 10 Hz). The injected carrier signal is tuned at *f_c_* = *f*_0_(θ = 90°) = 936.5 MHz. The output voltage waveform is plotted in Figure 17. It should be noted that, ideally, the envelope signal waveform should be the same as the time-dependent transmission coefficient of the S-SRR-loaded CPW. However, the envelope signal in practice depends on the readout system. Next, the envelope signal is converted into a digital rectangular signal to ease the signal processing of the readout system, and this conversion can be obtained by an electronic comparator. The measured angular velocity is 597.6 rpm (9.96 Hz), the error being 0.4%, in very good agreement with the nominal values. It is worth mentioning that in [27,28] the rotation speeds were set to 60 rpm and 3000 rpm, where the errors were 0.2% and 0.5%, respectively. Therefore, with the considered experimental set-up, the error systematically increases with the speed. Provided that the velocity is restrictedly constant, to enhance precision, the velocity measurement can be done between very distant nonconsecutive peaks (or by averaging the time between adjacent peaks).

Finally, it is important to highlight that, as long as the harmonic signal frequency is much higher than the linear frequency of rotation (this condition is always satisfied for practical frequencies), the measurable range of velocities is theoretically unlimited. Furthermore, a general discussion on the performance of the proposed sensing approach based on symmetry properties can be found in [27].

## 8. Conclusions

In conclusion, we have demonstrated that S-SRR-loaded CPW transmission lines are useful for the implementation of compact angular displacement and angular velocity sensors. Typically, the frequency of operation of microwave sensors is dictated by system requirements, EMI effects, EMC and/or crosstalk, or low-cost readout circuits. As frequently occurs in microwave circuits, the device size is mainly determined by the size of the passive structures, *i.e.*, the resonant element in the considered sensor. Thus, for miniaturization purposes, it is necessary to consider electrically small resonators, such as the S-SRR, which by virtue of its large inductance is electrically much smaller than other resonant particles which have been proposed for angular displacement and velocity sensors. The sensing principle is based on the controllability of line-to-resonator coupling through S-SRR rotation. Thereby a coupling-modulated resonance is obtained by rotation, which determines the magnitude of the attenuation peak. Hence, the relative angle between the S-SRR and the CPW is deduced by inspection of the peak magnitude. A prototype sensor has been validated by using a rotating cylinder (where the S-SRR was attached) controlled by a step motor. The characterization results indicate that the rotation sensor exhibits good linearity, sensitivity and dynamic range. The tests relative to the measurement of angular velocities indicate that the proposed approach is very accurate. A comparable performance as previously obtained by some of the authors using an ELC resonator is achieved, but the operation frequency is reduced to a half due to the electrically small size of the S-SRR.
